# Distributed Neural Activity Patterns during Human-to-Human Competition

**DOI:** 10.3389/fnhum.2017.00571

**Published:** 2017-11-23

**Authors:** Matthew Piva, Xian Zhang, J. Adam Noah, Steve W. C. Chang, Joy Hirsch

**Affiliations:** ^1^Interdepartmental Neuroscience Program, Yale School of Medicine, Yale University, New Haven, CT, United States; ^2^Department of Psychiatry, Yale School of Medicine, Yale University, New Haven, CT, United States; ^3^Department of Psychology, Yale University, New Haven, CT, United States; ^4^Department of Neuroscience, Yale School of Medicine, Yale University, New Haven, CT, United States; ^5^Department of Comparative Medicine, Yale School of Medicine, Yale University, New Haven, CT, United States; ^6^Department of Medical Physics and Biomedical Engineering, University College London, London, United Kingdom

**Keywords:** social interaction, temporal-parietal junction, functional near-infrared spectroscopy, hyperscanning, connectivity, coherence

## Abstract

Interpersonal interaction is the essence of human social behavior. However, conventional neuroimaging techniques have tended to focus on social cognition in single individuals rather than on dyads or groups. As a result, relatively little is understood about the neural events that underlie face-to-face interaction. We resolved some of the technical obstacles inherent in studying interaction using a novel imaging modality and aimed to identify neural mechanisms engaged both within and across brains in an ecologically valid instance of interpersonal competition. Functional near-infrared spectroscopy was utilized to simultaneously measure hemodynamic signals representing neural activity in pairs of subjects playing poker against each other (human–human condition) or against computer opponents (human–computer condition). Previous fMRI findings concerning single subjects confirm that neural areas recruited during social cognition paradigms are individually sensitive to human–human and human–computer conditions. However, it is not known whether face-to-face interactions between opponents can extend these findings. We hypothesize distributed effects due to live processing and specific variations in across-brain coherence not observable in single-subject paradigms. Angular gyrus (AG), a component of the temporal-parietal junction (TPJ) previously found to be sensitive to socially relevant cues, was selected as a seed to measure within-brain functional connectivity. Increased connectivity was confirmed between AG and bilateral dorsolateral prefrontal cortex (dlPFC) as well as a complex including the left subcentral area (SCA) and somatosensory cortex (SS) during interaction with a human opponent. These distributed findings were supported by contrast measures that indicated increased activity at the left dlPFC and frontopolar area that partially overlapped with the region showing increased functional connectivity with AG. Across-brain analyses of neural coherence between the players revealed synchrony between dlPFC and supramarginal gyrus (SMG) and SS in addition to synchrony between AG and the fusiform gyrus (FG) and SMG. These findings present the first evidence of a frontal-parietal neural complex including the TPJ, dlPFC, SCA, SS, and FG that is more active during human-to-human social cognition both within brains (functional connectivity) and across brains (across-brain coherence), supporting a model of functional integration of socially and strategically relevant information during live face-to-face competitive behaviors.

## Introduction

The dyadic neuroimaging literature thus far has established that cooperative and communicative behaviors are associated with across-brain coherence using a neuroimaging strategy called hyperscanning, which involves the imaging of two interacting individuals simultaneously (Montague et al., 2002). In an early hyperscanning study using functional magnetic resonance imagining (fMRI), subjects were required to perform a multi-round trust game with another subject while both were placed in separate scanners (King-Casas et al., 2005). The authors reported a temporal shift in the coordination of activity between middle and anterior cingulate cortex and dorsal striatum both within and across the brains of the interacting subjects that seemed to correlate with “intention to trust” (King-Casas et al., 2005). Recent studies exploring real-time social interaction between two or more individuals have begun to utilize functional near-infrared spectroscopy (fNIRS) due in part to its relative tractability in the simultaneous recording of multiple individuals using a single system. Functional NIRS allows individuals to perform interactive tasks face-to-face in ecologically valid contexts. One of the first of these studies required pairs of participants to either synchronize a button press, defined as cooperation, or to press the button faster than their partner, defined as competition (Cui et al., 2012). Increased coherence in the neural activity between partners was found in the superior frontal cortex only during cooperation (Cui et al., 2012). These findings were then independently replicated in a study that found divergent activity between female–female, male–male, and female–male pairs that seemed to correlate with differences in task performance (Cheng et al., 2015). This paradigm has more recently been used to show increased coherence in superior frontal cortex between lovers compared to between pairs of friends or strangers (Pan et al., 2017). Finally, other similar paradigms have shown increased synchrony between the dorsomedial prefrontal cortices of interacting partners specifically during cooperation (Liu et al., 2016).

Further studies using fNIRS to examine interpersonal interaction during communicative behaviors converge on frontal areas as well as the temporal-parietal junction (TPJ) as being preferentially coherent. During a communicative task between three subjects, interpersonal neural synchronization between the TPJ of leaders and followers was found to be higher than synchronization between two followers, suggesting that coherence between the TPJ regions of both subjects may underlie effective communication (Jiang et al., 2015). This corresponds to findings during a modified ultimatum game, in which synchronization between the TPJ regions of two partners was higher during face-to-face than back-to-back task performance (Tang et al., 2016). Other studies have also aimed to determine the significance of face-to-face visual access during social interaction. In a study that aimed to distinguish between face-to-face and back-to-back monolog and dialog conditions, coherence between left inferior frontal cortex was only found during face-to-face dialog, underscoring the importance of visual access to partners during communication (Jiang et al., 2012). Furthermore, recent work has dissected the importance of eye contact with a real partner as opposed to a picture of a face. In a recent study, coherence between specific across-brain region-pairs during eye-to-eye contact was taken as a measure of brain coupling that was specific to eye-contact with another person (Hirsch et al., 2017). It was found that the receptive functions associated with the canonical language areas were more synchronous during eye-to-eye contact than during mutual gaze to the eye region of a picture (Hirsch et al., 2017). These areas included the left middle and superior temporal gyri, the supramarginal gyrus (SMG), in addition to the pre-motor cortex (Hirsch et al., 2017).

Further insight into live interpersonal interaction can be gleamed from studies that examine neuropsychiatric disorders, such as autism spectrum disorder (ASD), which are characterized by deficits in social behavior marked by reduced mutual eye contact, among other symptoms (Senju and Johnson, 2009). Studying ASD patients through behavioral studies utilizing paradigms borrowed from behavioral economics and game theory have been informative in probing how this disorder can disrupt fundamental social behavior. For example, ASD patients display a diminished capability to differentiate between cooperative and uncooperative partners in a simulated ball game compared to typically developing control subjects (Andari et al., 2010). Furthermore, ASD patients tend to be less influenced by the presence of other individuals when deciding whether to make generous or selfish decisions in a dictator game (Izuma et al., 2011) and display further atypical inferences about opponents in the stag hunt game (Yoshida et al., 2010). These studies further elucidate how interactive social behaviors can be impacted by disruptions to typical socio-cognitive development.

However, such studies examining interpersonal competition remain sparse. In a previous fMRI investigation that involved neuroimaging of a single participant, subjects were asked to play a simplified poker game against either a human opponent or a preprogrammed computer opponent (Carter et al., 2012). The authors then utilized a region of interest (ROI) approach with multi-voxel pattern analysis (MVPA) to identify regions particularly important when playing against a human opponent (Carter et al., 2012). Using these methods, the authors determined that the TPJ was uniquely involved in playing a human rather than computer opponent compared to all other ROIs examined (Carter et al., 2012). A later meta-analysis from the same group indicated that multiple information streams, including attention, memory, and language systems, converge at the TPJ, which may function as a hub for integrating socially relevant information (Carter and Huettel, 2013). Additionally, when the TPJ was separated into its two anatomically distinct components, SMG and angular gyrus (AG), the two areas were found to be largely associated with distinct processes, with SMG more related to attention reorienting and AG more related to theory of mind (Carter and Huettel, 2013).

**FIGURE 1:**
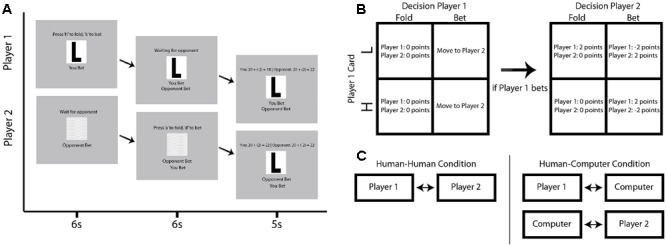
Schematic of experimental task. **(A)** An example progression through a single trial in which Player 1 is given a Low card and both players bet. Top row of screens indicates the progression of Player 1, while the bottom row of screens indicates the progression of Player 2. Each column indicates a discrete subsection of the example trial, lasting 6, 6, and 5 s, respectively. **(B)** Multistep contingency table showing the possible outcomes for each potential trial type. The top row indicates contingencies for when Player 1 receives a Low card, while the bottom row indicates the contingencies for when Player 1 receives a High card. In the right table, the columns indicate whether Player 1 bets or folds. If Player 1 bets, Player 2 is allowed to bet or fold in response, with contingencies indicated by the left table. **(C)** Diagram describing human–human and human–computer conditions. Participants are in the exact same location in each condition, but playing each other in the human–human condition and concurrently playing computer opponents in time-synchronized but distinct games in the human–computer condition.

In order to extend these findings, we developed a bidirectional (two-person) version of the poker game with simultaneous fNIRS recordings from pairs of participants (**Figures 1A,B**). The participants played the simplified poker game face-to-face in two separate conditions (**Figure 1C**). In one condition, participants played the poker game against each other (the human–human condition). In a second condition, the participants played the poker game simultaneously against computer opponents (the human–computer condition). Within-brain functional connectivity analyses were used to test the hypothesis that greater connectivity would be observed between discrete neural structures in the human–human condition than in the human–computer condition. We hypothesized that areas involved with social cognition, particularly the TPJ, would increase functional connectivity with prefrontal strategy centers when humans played against conspecifics rather than computer opponents. We further hypothesized that measures of across-brain coherence would be higher in the human–human condition than in the human–computer condition for pairs of structures implicated in previous studies of social cognition. Wavelet analysis (Compo and Torrence, 1998) was used to determine across-brain coherence signatures in both conditions and introduced a novel measure of across-brain coherence between two interacting subjects.

## Materials and Methods

### Participants

A total of 20 pairs of subjects participated in this study after being recruited from the Yale and Gateway Community College campuses (age: 25.38 ± 4.9 years, range 18–41 years). None of these subjects were associated with our laboratory in any capacity. Out of the pairs, 2 were male–male, 4 were female–female, and 16 were male–female. All but 3 of the subjects were right-handed, and all had corrected-to-normal vision. Subjects rated their expertise in poker an average of 2.1 ± 0.1 points out of 5 (range 1–3 points). Subjects rated their familiarity with their opponent an average of 1.9 ± 0.2 points out of 5 (range 1–5 points). Consistent with reports that dyads were largely either strangers or acquainted only as students, 75% of subjects selected 1 to describe their familiarity with their opponent, indicating that they were complete strangers. Of the remaining 25%, scores ranged from 2 to 5, with 15% indicating a score greater than 3. Familiarity was not found to correlate with any of the relevant behavioral or neural measures examined, and a reanalysis of across-brain coherence results excluding all pairs of subjects who were not complete strangers did not change the observed results (Supplementary Figure S1). Written informed consent was obtained from each participant in accordance with study procedures approved by the Institutional Review Board of the Yale University School of Medicine (HIC #1512016895).

Prior to the experimental task, each subject was required to perform a simple right-handed finger-thumb tapping task to validate the quality of the neural signals recorded. Task-related activity in left primary motor cortex was a requirement for eligibility in this study, confirming the quality of neural signals. All subjects who met the eligibility requirements were included in the data set, and no subjects were excluded following acquisition.

### Tasks

Subjects sat across from each other in plain view with a computer screen to their side to play a simplified poker game (Rapoport et al., 1997; Carter et al., 2012) in each of two separate conditions. Responses were made with a keyboard. The rules of each condition were identical. On any given trial, subjects were either given the designation of Player 1 or Player 2. These roles switched every trial, with a total of 40 trials per condition. At the outset of each condition, both players were given 20 points. At the beginning of each trial, Player 1 was randomly given either a “High” or a “Low” card. Player 1 then had 6 s to decide whether to bet and continue with the trial or fold and end the trial without losing or gaining any points in response to the given card. As with the remaining stages of each trial, both players were required to wait until the end of the full 6 s for the trial to progress regardless of their reaction time. At the end of this 6 s, Player 2 was then able to bet or fold in response to the action of Player 1. Since Player 2 was not dealt a card, his or her objective was to determine what card the opponent was dealt, as this would determine the outcome of the trial. At the end of this second round of 6 s, a results screen was shown for 5 s, revealing the card, the points gained or lost during the trial, and the running score of both participants (**Figure 1A**).

If Player 1 decided to fold, Player 2 could not bet and could only accept Player 1’s fold. Both players then neither gained nor lost any points. If Player 1 decided to bet, then Player 2 had the option to either bet or fold in response to the decision of Player 1. If Player 2 bet and Player 1 had a Low card, Player 2 won 2 points and Player 1 lost 2 points. If Player 2 bet and Player 1 had a High card, Player 2 lost 2 points and Player 1 won 2 points. If Player 2 folded, Player 2 neither gained nor lost any points, regardless of what card Player 1 was dealt. If Player 2 folded and Player 1 had a Low card, this was a successful bluff, and Player 1 won 2 points. If Player 1 had a high card and Player 2 folded, Player 1 had not successfully bluffed Player 2, and Player 1 neither gained nor lost any points. See **Figure 1B** for a summary of these contingencies.

This game was played in two different conditions. In one condition, subjects played against each other. In the second condition, subjects played simultaneously against computer opponents. However, they remained seated across from each other as in the first condition. These computer opponents were identically programmed to play with a fixed optimal strategy and did not shift their strategy based on the participants’ responses. Moreover, the timing was matched for both the human–human and human–computer conditions. Participants were told explicitly whether they were playing against human or computer opponents just prior to the start of each condition. The human–human condition was completed first for half of participants, while the human–computer condition was completed first for the other half.

Subjects were told prior to completing the two conditions that they would receive 15 USD for their participation in the study, with a possibility of earning up to 5 USD in bonus money contingent upon their score for both conditions. This was meant to ensure that participants remained engaged with the task. Although no subject scored high enough to earn the full 5 USD bonus according to the information given before task completion, all subjects were given 20 USD at the conclusion of the study regardless of performance. Following completion of the experimental tasks, subjects were asked to rate the difficulty of the human vs. computer opponent on a five-point scale.

### Data Collection

#### Functional NIRS Signal Acquisition

Using a 64-fiber Shimadzu LABNIRS system (Shimadzu Corporation, Kyoto, Japan), measurements of oxygenated hemoglobin (Hbo) and deoxygenated hemoglobin (Hbr) were acquired simultaneously from each subject in a given pair during task performance. Customized caps were used for optode placement, with 42 channels divided into two hemispheres for each individual subject. Distance between emitter and detector pairs was 3 cm, and signals were acquired at a sampling interval of 27 ms. Hair was removed from the optode channels prior to optode placement with a lighted fiber-optic probe (Daiso, Hiroshima, Japan). Resistance for each channel prior to reading was determined to ensure acceptable signal-to-noise ratios for each channel, with adjustments made as needed to meet minimum criteria defined in the LABNIRS recording software (Tachibana et al., 2011; Ono et al., 2014; Noah et al., 2015).

The continuous lasers emit 3 wavelengths of light at 780, 805, and 830 nm, and detectors measure changes in Hbo and Hbr concentrations. At each individual channel, absorption of light in tissue for the three wavelengths is converted into the appropriate hemoglobin concentrations via a modified Beer–Lambert equation, with raw optical density changes converted into relative chromophore concentration changes (i.e., ΔHbo, ΔHbr, and ΔTotalHb) based on the following equations (Cope et al., 1988; Matcher et al., 1995; Boas et al., 2004):

$$\begin{array}{c} \Delta Hbo\;=\;-1.4887\;\times \;\Delta abs780\;+\;0.5970\;\times \;\Delta abs805\;+\;1.4847\;\times \Delta abs830;\\ \Delta Hbr\;=\;1.8545\;\times \;\Delta abs780\;+\;(-0.2394)\;\times \;\Delta abs805\;+(-1.0947)\;\times \;\Delta abs830;\\ \Delta TotalHb\;=\;\Delta Hbo\;+\;\Delta Hbr. \end{array}$$

In the above equation, the additional terms refer to absorbance of light at 780, 805, and 830 nm, respectively.

#### Optode Localization

A Patriot 3D Digitizer (Polhemus, Colchester, VT, United States) with previously described linear transform techniques (Okamoto and Dan, 2005; Singh et al., 2005; Eggebrecht et al., 2012; Ferradal et al., 2014) was used to determine the anatomical coordinates of all channels in relation to standard anatomical landmarks including inion, nasion, Cz, and left (T3) and right (T4) tragi. The MNI coordinates for the channels were obtained using NIRS_SPM software (Ye et al., 2009) with MATLAB (Mathworks, Natick, MA, United States), and the corresponding anatomical locations of each channel were determined by the atlas provided (Damasio and Damasio, 1989; Rorden and Brett, 2000). Group-averaged MNI coordinates for all channels and group-averaged anatomical locations are listed in Supplementary Table S1.

### Signal Processing

#### Preliminary Processing

Baseline drift was modeled via detrending using a polynomial of the fourth degree:

$$\begin{array}{c} P(t)\;=\;a_{0}\;+\;a_{1}t\;+\;a_{2}t^{2}\;+\;a_{3}t^{3}\;+\;a_{4}t^{4}, \end{array}$$

which was fitted to the raw fNIRS signal. The difference between the raw data and the polynomial model was taken as the detrended data. Channels lacking signal were identified automatically and then confirmed with visual inspection based on the following criteria: (1) positive correlation between Hbo and Hbr signals, corresponding with basic physiology, and (2) a magnitude of over 10 times the average signal for each individual subject. These criteria resulted in approximately 5% of signals being removed prior to further analysis.

Independent components analysis (ICA) was employed for automatic spike removal (Delorme and Makeig, 2004). Spikes were defined by the following criteria: (1) accumulated data points of all spikes were less than 1% of the total data, and (2) the peak amplitude of the spike exceeded four standard deviations of the entire time series of the IC. Functional NIRS data were down-sampled for an effective rate of 0.81 s and were reshaped into 4 × 4 × 223 images. Hbr data were used in further analyses as the proxy for neural activity due to the reduced sensitivity to systemic artifacts and global effects (Zhang et al., 2016).

#### Global Mean Removal

Blood pressure, respiration, blood flow variation, and other global systemic effects have been shown to impact measured neural signals (Kirilina et al., 2012; Tak and Ye, 2014). The global signal components are assumed to reflect these systemic effects and were removed using a PCA-spatial filter (Zhang et al., 2016) prior to analyses using general linear model (GLM), psychophysiological interaction (PPI), and wavelet coherence techniques. This technique exploits the extensive head coverage of our optode placement and distinguishes between signals that are locally generated vs. globally distributed.

### Data Analysis

#### Behavioral Analyses

Behavioral variables including reaction times and decision proportion in the poker game were analyzed using two-way ANOVAs with experimental condition and task situation as factors. Differences within individual task situations between the human–human and human–computer conditions were compared with Tukey-Kramer *post hoc* tests. Task performance of humans playing against human opponents, humans playing against computer opponents, and computers playing against human opponents were compared with a one-way ANOVA using player type as a factor. Self-rated difficulty of the human–human and human–computer conditions was compared using a paired-sample *t*-test. For the behavioral influence analysis, cross-correlation coefficients were determined to describe the influence of an opponent’s previous decision on a given player’s current decision. These cross-correlation coefficients were calculated separately for the human–human and human–computer conditions, converted into Fisher’s *z*, and compared using a paired-sample *t*-test.

#### Functional Connectivity

Psychophysiological interaction (PPI) analysis was performed via the gPPI toolbox in SPM8 (Mclaren et al., 2012). This analysis follows from the assumption that neural activity as inferred from the canonical hemodynamic response function (HRF) can be estimated via a deconvolution method (Friston et al., 2003). We modeled the 6-s period in which subjects were allowed to make their decision and ultimately performed a contrast between the human–human and human–computer conditions. Supramarginal gyrus (SG) and AG, both components of the canonical TPJ, were chosen as seeds. While these brain areas have consistently been implicated in studies of social cognition (Van Overwalle, 2009), an influential study using a poker paradigm analogous to our task found that the TPJ was specifically and uniquely implicated when playing against a human rather than computer opponent (Carter et al., 2012). A meta-analysis concerning the potentially unique role the TPJ plays in social cognition indicated that the TPJ may function as a hub for multiple streams of socially relevant information (Carter and Huettel, 2013). Additionally, the meta-analysis indicated divergent roles for SG and AG, two anatomically distinct components of the TPJ. Specifically, while SG was found to be implicated in attention reorienting, AG was found to be more implicated in theory of mind (Carter and Huettel, 2013). More recent fNIRS studies have also converged on the TPJ as relevant in dyadic neuroimaging paradigms (Jiang et al., 2015; Tang et al., 2016). Therefore, we decided to utilize two distinct seeds, one for SG and one for AG. We hypothesized that AG would uniquely connect with prefrontal centers known to be important in strategic decision-making, potentially aligning with its unique role in theory of mind processes (Carter and Huettel, 2013).

#### Contrast Effects

Contrasts between conditions (human–human vs. human–computer) were performed by channel-wise comparisons. All channel locations for each subject were converted to MNI space, and registered to median locations using non-linear interpolation. Once in normalized space, comparisons across conditions were based on discrete channel units. A channel-wise GLM analysis was performed to contrast the 6-s decision-making time period between the human–human and human–computer conditions.

#### Across-Brain Coherence

Coherence across the brains of dyads was determined as a function of frequency and time. Time series of the neural signals were decomposed into multiple frequency components using wavelet analysis (Compo and Torrence, 1998), with coherence between these corresponding components based on a complex conjugation (Cui et al., 2012). Wavelet analysis can be thought of as the local correlation of two time series and can thus be more effective than more traditional time series analyses in elucidating phase-locked signals (Cui et al., 2012). Coherence across time was averaged across subjects at each frequency and compared between the human–human condition and human–computer condition with a permutation test. Similar analyses have been used with fNIRS previously to study interpersonal interaction (Cui et al., 2012; Jiang et al., 2012; Scholkmann et al., 2013). For more thorough explanations of this technique along with illustrative examples, see previous work on this topic, including pioneering studies in geophysics (Grinsted et al., 2004) and fMRI resting state (Chang and Glover, 2010). To determine whether across-brain coherence effects were specific to interacting partners, coherence was also calculated between mismatched subjects (“scrambled” partners). In this analysis, each subject was paired with every other participant except the original partner, and coherence in both the human–human and human–computer conditions was calculated and compared.

## Results

### Behavioral Differences between Conditions Reflect the Importance of Immediate Choice History

**FIGURE 2:**
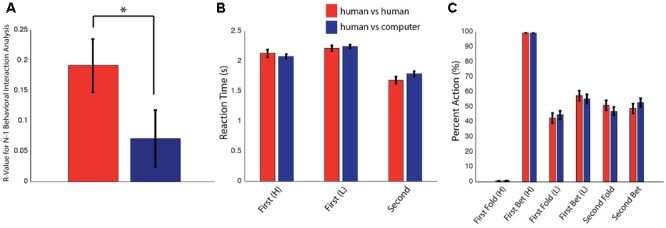
Behavioral results. Data are presented as mean ± SEM. ^∗^*P* < 0.05. **(A)** Cross-correlation coefficients for the effect of an opponent’s immediately previous decision on a player’s current decision separated for the human–human and human–computer conditions. These values were determined to be significantly different after conversion to *z*-scores with a paired *t*-test. **(B)** Reaction times for Player 1 with a High or Low card and for Player 2 in the human–human and human–computer conditions. No significant differences were observed between the two conditions. **(C)** Betting and folding behavior for Player 1 with a High or Low card and for Player 2 in the human–human and human–computer conditions. Players in both conditions seemed to play to the optimal overall strategy. No significant differences were observed between conditions.

The impact of an opponent’s previous decision on a given player’s current decision as determined using an *N*-1 analysis indicated that *z*-scores of the cross-correlation coefficients in the human–human condition (*r* = 0.19) and the human–computer condition (*r* = 0.07) varied [**Figure 2A**; cross-correlation coefficients calculated between an opponent’s immediately previous decision and a player’s current decision shown for the human–human and human–computer conditions on the *y*-axis; *t*(38) = 2.1, *P* = 3E-02, paired-sample *t*-test]. This demonstrated that subjects were more influenced by an opponent’s previous decision when playing against human rather than computer opponents and thus indicated a difference in the influence of a human opponent on a given player’s decision. Importantly, the human–human and human–computer conditions were matched for all other values tested. Reaction times and overall choice behavior were not different across the two conditions for any game state (**Figures 2B,C**; averaged reaction times and percent folding or betting for the human–human and human–computer conditions for all possible game states on the *y*-axis). Moreover, scores across all conditions were matched, as subjects scored similar amounts of points against both human participants and computer opponents and did not outperform the computer as a group (human vs. human: 27.1 ± 1.3 points, human vs. computer: 28.2 ± 1.3 points, computer: 27.2 ± 1.8 points). Self-reported difficulty of human vs. computer opponents was also not significantly different (human difficulty: 2.6 ± 0.2, computer difficulty: 2.4 ± 0.2). Together, these behavioral results indicate that differences between conditions were limited to effects of immediate choice history and not to differences in overall implemented strategy or difficulty between conditions.

### Functional Connectivity and Contrast Effects Are Modulated Depending on Opponent Type

**FIGURE 3:**
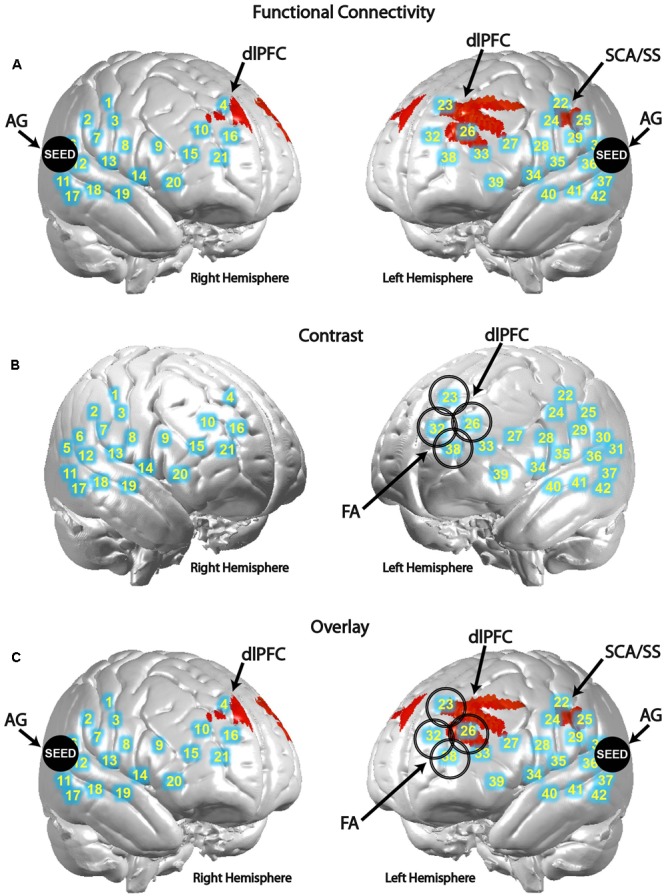
Angular gyrus (AG) functional connectivity and observed contrast effects indicate differences between conditions. Blue circles with corresponding numbers show average channel locations. **(A)** Black filled circles indicate centroids of seed regions, bilateral AG (MNI coordinates: ±57, 70, 26). Red areas show regions with higher functional connectivity to the seed during the human–human condition over the human–computer condition in SCA and dorsolateral prefrontal cortex [*P* < 0.01; left clusters, MNI coordinate of peak voxel -64, -10, 32, *t*(25) = 3.16, *P* = 2E-03, SCA = 0.45, SS-BA2 = 0.16, SS-BA1 = 0.12; MNI coordinate of peak voxel -30, 22, 40, *t*(25) = 2.97, *P* = 3E-03, dlPFC-BA9 = 1.00; MNI coordinate of peak voxel -26, 44, 22, *t*(25) = 2.72, *P* = 6E-03, dlPFC-BA46 = 1.00; MNI coordinate of peak voxel -34, 48, 24, *t*(25) = 2.60, *P* = 8E-03, dlPFC-BA46 = 0.82, pars triangularis Broca’s area = 0.18; right cluster, MNI coordinate of peak voxel 6, 48, 36, *t*(25) = 2.58, *P* = 8E-03, dlPFC-BA9 = 1.00]. **(B)** Larger open black circles show the location of channels increased activity in the human–human condition relative to the human–computer condition [*P* < 0.05; channel 23, MNI coordinate -16, 48, 47, *t*(39) = 2.67, *P* = 5E-03; channel 26, MNI coordinate -33, 49, 32, *t*(39) = 1.70, *P* = 5E-02; channel 32, MNI coordinate -17, 62, 30, *t*(39) = 2.58, *P* = 7E-03; channel 38, MNI coordinate -29, 63, 15, *t*(39) = 2.21, *P* = 2E-02]. **(C)** Overlay of functional connectivity and contrast effects.

Differences in functional connectivity within individual subjects during the poker game were determined by using areas identified in previous research as seeds in a PPI analysis, including SMG and AG. Both are components of the canonical TPJ, implicated in previous studies to be preferentially recruited when playing against a human rather than a computer opponent in a similar poker paradigm (Carter et al., 2012). Although SMG showed no significant connectivity findings, AG showed greater connectivity with left subcentral area (SCA), left somatosensory cortex (SS), and bilateral dorsolateral prefrontal cortex (dlPFC) in the human–human condition relative to the human–computer condition (**Figure 3A** and **Table 1**). These findings are consistent with our hypothesis that AG would be more involved in our task due to its potential role in theory of mind processes (Carter and Huettel, 2013). In a channel-wise GLM analysis, increased activity was observed when contrasting the human–human condition over the human–computer condition in a discrete cluster of channels centered on left frontopolar area (FA) and dlPFC (**Figure 3B** and **Table 2**). Degrees of freedom vary between analyses because the number of usable channels differed with some subjects due to anatomical variability related to head size. These overlapping findings between functional connectivity and contrast effects (**Figure 3C**) represent the first indication of functional connectivity between areas implicated in social (AG) and strategic (dlPFC) cognition during an interactive social task. The functional connectivity of AG with SCA/SS corresponds to recent work regarding the neural correlates of live eye-to-eye contact between partners, suggesting that SCA/SS may be implicated in dynamic social exchanges (Hirsch et al., 2017).

**Table 1:** Functional connectivity [human–human] > [human–computer].

Analysis	Coordinates^∗^	Peak voxels *t*-value (degrees of freedom)	*P*	Anatomical regions in cluster	BA	Probability	*n* of voxels
PPI with angular gyrus	(–64, -10, 32)	3.16 (25)	0.002	Subcentral area	43	0.45	251
(±57, -70, 26) as seed				Primary somatosensory cortex	1, 2	0.28	
	(–30, 22, 40)	2.97 (25)	0.003	Dorsolateral prefrontal cortex	9	1.00	324
	(–26, 44, 22)	2.72 (25)	0.006	Dorsolateral prefrontal cortex	46	1.00	39
	(–34, 48, 24)	2.60 (25)	0.008	Dorsolateral prefrontal cortex	46	0.82	10
				Pars triangularis Broca’s area	45	0.18	
	(6, 48, 36)	2.58 (25)	0.008	Dorsolateral prefrontal cortex	9	1.00	8

*Functional connectivity increases determined by psychophysiological interaction (PPI) analysis in the human–human condition relative to the human–computer condition are shown for the anatomically defined AG seed. Areas included in the functional networks are identified by MNI coordinates, levels of statistical significance (*t*-value and *P*), regions included in the cluster including Brodmann’s Area (BA), estimated probability, and *n* of voxels, which is a measure of cluster volume.*

*^∗^Coordinates are based on the MNI system and (-) indicates left hemisphere. Seed centroids are based on channel groups for which coordinates from right and left hemisphere clusters have been averaged. Averages account for minor inter-individual variations in channel numbers for each group. Probability refers to the probability of a certain cluster corresponding to the listed neural structure as evaluated by the MNI atlas.*

**Table 2:** Channel-wise contrast effects and functional connectivity [human–human] > [human–computer].

Analysis	Channel number	Coordinates^∗^	*t*-value (degrees of freedom)	*P*	Anatomical region	BA	Probability
Channel-wise GLM	23	(–16, 48, 47)	2.67 (39)	0.005	Dorsolateral prefrontal cortex	9	1.00
	26	(–33, 49, 32)	1.7 (39)	0.049	Dorsolateral prefrontal cortex	46	0.84
					Dorsolateral prefrontal cortex	9	0.15
	32	(–17, 62, 30)	2.58 (39)	0.007	Frontopolar area	10	0.56
					Dorsolateral prefrontal cortex	9	0.28
					Dorsolateral prefrontal cortex	46	0.16
	38	(–28, 63, 15)	2.21 (39)	0.016	Frontopolar area	45	0.75
					Dorsolateral prefrontal cortex	46	0.25

*Contrast effects determined by general linear model (GLM) analysis for the human–human condition over the human–computer condition are shown. Significant channels are identified by channel number, MNI coordinates, levels of statistical significance (*t*-value and *P*), regions corresponding to the channel including Brodmann’s Area (BA), and estimated probability.*

*^∗^Coordinates are based on the MNI system and (-) indicates left hemisphere. Probability refers to the probability of a certain cluster corresponding to the listed neural structure as evaluated by the MNI atlas.*

### Across-Brain Coherence Differs between Conditions

**FIGURE 4:**
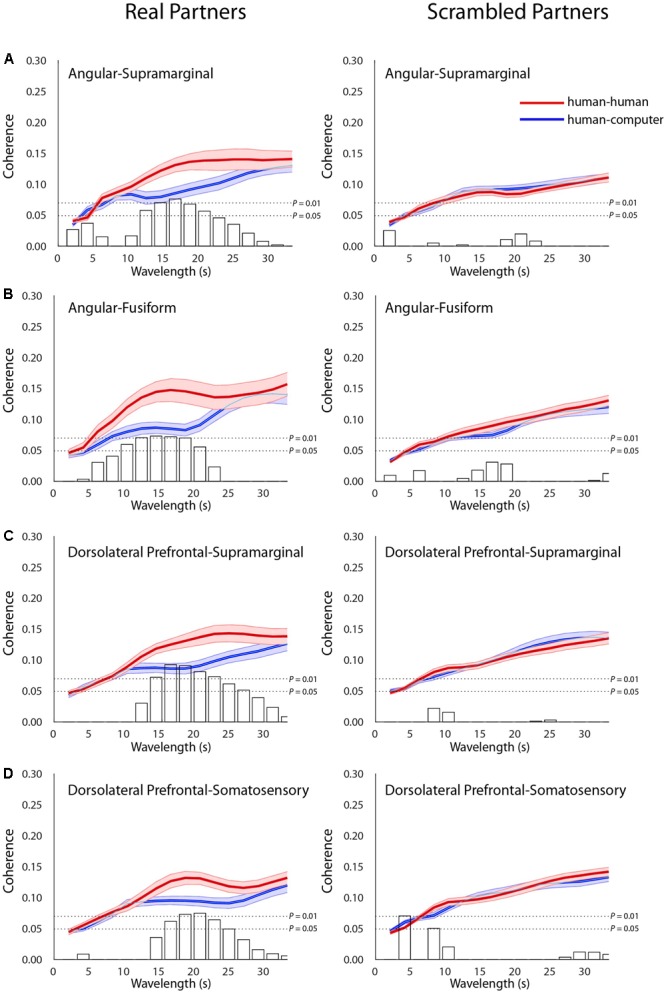
Across-brain coherence varies as a function of condition between distinct neural structures. Signal coherence between subjects (*y*-axis) is plotted against the wavelength (*x*-axis) for the human–human (red) and the human–computer (blue) conditions (shaded areas: ±1 SEM). Bar graphs indicate the calculated *t*–values for the contrast between the two conditions for each of the wavelength values. The upper horizontal dashed line indicates *P* = 0.01 and the lower line indicates *P* = 0.05, as determined via permutation test. Left panels show coherence between actual partners, and right panels show coherence between scrambled partners. Across-brain coherence is shown between **(A)** Angular gyrus and supramarginal gyrus [Partners: *t*(27) = 3.02, *P* = 6E-03; Scrambled: no significant effect], **(B)** Angular gyrus and fusiform gyrus [Partners: *t*(26) = 2.90, *P* = 7E-03; Scrambled: no significant effect], **(C)** dorsolateral prefrontal cortex (BA46) and supramarginal gyrus [Partners: *t*(35) = 3.60, *P* = 1E-03; Scrambled: no significant effect], and **(D)** dorsolateral prefrontal cortex (BA46) and somatosensory cortex [Partners: *t*(39) = 2.86, *P* = 6E-03; Scrambled: no significant effect]. Degrees of freedom vary because the number of usable channels differed with some subjects due to anatomical variability related to head size and bad channels.

In addition to connectivity and contrast findings within individual subjects, we hypothesized that neural measures characterizing the interaction between subjects will vary between the human–human and human–computer conditions, providing a potential neural substrate for the differences in decision-making observed between conditions. Signals related to human–human and human–computer gameplay were decomposed into frequency components. This effectively removes low-frequency task effects and reveals fine-grain temporal oscillations employed to compare synchrony across partners. Temporal period (*x*-axis, seconds) and across-brain coherence (*y*-axis, correlation) functions were determined for the human–human (red) and human–computer (blue) conditions (**Figure 4**). A complex of neural structures increased coherence in the human–human condition for specific wavelengths, including AG with SMG [**Figure 4A**; *t*(27) = 3.02, *P* = 6E-03], AG with fusiform gyrus (FG) [**Figure 4B**; *t*(26) = 2.90, *P* = 7E-03], SMG with dlPFC [**Figure 4C**; *t*(35) = 3.60, *P* = 1E-03], and dlPFC with SS [**Figure 4D**; *t*(39) = 2.86, *P* = 6E-03, permutation tests]. Degrees of freedom vary because the number of usable channels differed with some subjects due to anatomical variability related to head size. Because differences in familiarity between pairs of subjects could be an especially pervasive confounding factor for these across-brain analyses, we repeated these analyses using only subjects that were complete strangers. All results held when the data were reanalyzed, despite a reduced sample size (Supplementary Figure S1). Notably, as both AG and SMG are components of the canonical TPJ, these findings are the first to show across-brain TPJ coherence with areas involved in the visual recognition of faces, FG and SS (Adolphs et al., 1996, 2000; Kanwisher et al., 1997), and the implementation of strategic behaviors and working memory, dlPFC (Macdonald et al., 2000; van ’t Wout et al., 2005; Steinbeis et al., 2012), during an interactive social task. Crucially, when the neural data from each pair of participants were scrambled such that the data were compared between participants who did not perform the task concurrently, significant differences between conditions were not observed (**Figures 4A–D**). This indicated that across-brain correlations were due to events related to the temporal structure of the interaction between a set of partners, and not to operations that could be generalized to mismatched pairs. Additionally, findings were specific to the structures listed above, as no other pairs of structures were distinguished to the same level of significance between the human–human and human–computer conditions. These results implicate a network of structures interacting across brains that are preferentially recruited during interaction with a human opponent, strongly corresponding to the areas implicated in single-brain analyses of activation and functional connectivity.

## Discussion

We hypothesized that during naturalistic two-person social competition the TPJ would form a within- and across-brain network with prefrontal areas, functionally connecting neural structures important in both social cognition and strategic decision-making. Thus, this study aimed to elucidate the integrated and distributed neural effects underlying interpersonal competition through a bidirectional version of a simplified poker game played either with human or computer opponents. Behavioral results indicated differences between the effects of immediate choice history when interacting with human rather than computer opponents, while other measured variables, including overall choice behavior, reaction times, performance, and self-rated difficulty, were not different. Neural differences in patterns of activity and functional connectivity within brains as well as coherence across two interacting brains could represent the neural correlates of the specific difference in the influence of immediate choice history observed between the human–human and human–computer conditions. Multiple analyses of neural data resulted in an overlapping set of structures previously implicated in the cognition required for successful interactive game-play with a human opponent (Carter et al., 2012). While functional connectivity (PPI analysis) using AG, a component of TPJ, as a seed indicated stronger functional connectivity with SCA/SS and dlPFC, GLM comparisons correspondingly showed more activity in dlPFC/FA while subjects played human rather than computer opponents. These findings integrate TPJ-related social cognition systems (Carter et al., 2012; Carter and Huettel, 2013) with dlPFC strategic and executive control systems (Macdonald et al., 2000; van ’t Wout et al., 2005; Steinbeis et al., 2012) specifically during interactive game-play against human opponents. Additionally, across-brain coherence was observed in overlapping structures, including SMG, FG, and SS. Both FG and SS have been implicated in the recognition and evaluation of faces (Adolphs et al., 1996, 2000; Kanwisher et al., 1997), and the finding that both are involved in a coherent network of structures across subjects suggests that the involvement of sensing and recognizing facial expressions in others is involved at the neural level with structures noted in strategic and social cognition. Recent studies in both non-human primates (Dal Monte et al., 2016) and human subjects (Hirsch et al., 2017) have indicated unique behavioral and neural signatures associated with eye-contact with a real-life conspecific, overlapping with those identified in this study. Specifically, a direct contrast involving eye-to-eye contact with a real partner over eye-to-eye contact with a picture indicated greater activity in SCA/SS, potentially indicating its importance during in-person social interaction (Hirsch et al., 2017). The indication of greater functional connectivity between AG and SCA/SS during competition with a human rather than computer opponent further indicates the importance of this area during social interaction with a real-life conspecific. Together, these findings provide a potential framework for a complex of neural structures existing both within and across brains, specialized for the integration of multiple streams of relevant information.

Our observation that differences between playing against a human vs. a computer opponent are specific to a difference in the influence of immediate choice history is consistent with findings from previous versions of the simplified poker game (Carter et al., 2012). Our neural findings are likewise consistent with TPJ serving as a hub for multiple information streams associated with social cognition (Carter and Huettel, 2013). The connectivity data presented extend previous findings by suggesting that the two canonical components of TPJ, AG and SMG, either functionally connect to prefrontal strategy centers within individual brains and/or cohere with key visual and sensory areas across brains, perhaps underlying predictions of opponent behavior more relevant when playing a human opponent than a computer. Through this insight into the role that TPJ plays in a larger network of cortical structures spanning two interacting subjects, our results advance knowledge of the cortical underpinnings of social behaviors. While informative, previous studies have been limited to single-brain paradigms utilizing techniques specialized in detecting activity in individual brain areas. We have utilized an interactive paradigm with concurrent neural recordings and analytics specialized to detect the coordination of multiple neural areas both within a single brain and across two interacting brains.

Thus, fNIRS is a uniquely suited neuroimaging modality for our paradigm, compared to fMRI and electroencephalography (EEG) approaches. As with fMRI, observed variation in blood oxygenation serves as a proxy for neural activity when using fNIRS (Kato, 2004; Ferrari and Quaresima, 2012; Scholkmann et al., 2013; Gagnon et al., 2014). Although fNIRS has been used extensively in infant and child neuroimaging studies as opposed to largely incompatible fMRI approaches (Bortfeld et al., 2009; Kita et al., 2011; Lloyd-Fox et al., 2011; Xiao et al., 2012; Farroni et al., 2013; Iwanaga et al., 2013; Keehn et al., 2013; Kikuchi et al., 2013; Fava et al., 2014; Yasumura et al., 2014; Filippetti et al., 2015), fNIRS has not been widely applied to adult cognitive research until recently (Cutini et al., 2012) with exciting new directions including usage with immersive virtual reality (Seraglia et al., 2011). Additionally, recent adaptations of fNIRS for hyperscanning, including full-head coverage of detectors and removal of systemic artifacts (Zhang et al., 2016), enable significant advances in the neuroimaging of neural events that underlie interactive and social functions. Although it is well documented that signals acquired by fMRI and fNIRS are highly correlated (Strangman et al., 2002; Sato et al., 2013), the acquired signals are not identical. Two signals, oxyhemoglobin (Hbo) and deoxyhemoglobin (Hbr), are acquired by fNIRS, with the Hbr signal most closely resembling the fMRI signal (Sato et al., 2013). The fNIRS signals originate from larger volumes than fMRI signals, limiting spatial resolution (Eggebrecht et al., 2012). However, the fNIRS signal is acquired at a higher temporal resolution than the fMRI signal (20–30 ms vs. 1.0–1.5 s) and thus benefits studies of the neural connectivity within individual brains and coherence across brains (Cui et al., 2011; Sasai et al., 2012), although temporal resolution of task events is still limited by the speed of the physiological processes related to blood recruitment mechanisms. Finally, the sensitivity of fNIRS is limited to relatively superficial cortical structures within 2–3 cm from the skull surface. Fortunately, these limitations do not significantly impact our overall study aims, as some regions of interest that are involved with social cognition are located superficially in the cerebral cortex and accessible using fNIRS. Additionally, fNIRS has the added advantage of allowing subjects to sit face-to-face while performing interactive social tasks, as opposed to being located in isolated, separate scanners. It is important to note that EEG techniques have been used successfully in naturalistic hyperscanning studies in the past (Babiloni and Astolfi, 2014), with recent studies even taking place directly in classrooms (Dikker et al., 2017). However, fNIRS has the added advantage of accurate source localization of neural signals, which remains a significant challenge with EEG (Wendel et al., 2009). Together, these factors indicated to us that fNIRS was the best choice for our study given our experimental aims and paradigm.

When considering coherence of neural activity across two brains, it is possible for the observed effects to be driven by extraneous features of the experimental task, such as coordinated motor actions or perception of similar visual stimuli. Control for these factors included keeping the timing of gameplay and access to visual stimuli consistent between the human–human and human–computer conditions. Importantly, no differences between conditions were noted when partners were scrambled, indicating that effects were likely due to the temporal structure of the task uniquely occurring between partners and not to events independent of a shared temporal structure that are common even to mismatched partners. Previous work has indicated that fNIRS signals can be confounded by a global response to task demands, which can be overlooked when only specific neural regions are sampled (Zhang et al., 2016). To overcome this potential shortcoming of our neuroimaging technique, we utilized the Hbr signal, which corresponds more closely to the hemodynamic response observed in fMRI and is less susceptible to the global mean artifact in fNIRS (Sato et al., 2013; Zhang et al., 2016). Another unavoidable limitation of fNIRS is the inability to sample activity from deep neural areas, including limbic and striatal structures, which could have been informative in our dual-brain paradigm. We note that although functional connectivity effects were observed with TPJ, contrast effects did not reveal segregated activity in these regions. A likely explanation for this discrepancy between the fMRI findings (Carter et al., 2012) and the fNIRS findings of this study relates to the limitation of sensitivity greater than 3 cm below the cortical surface.

Additional limitations of our study involve our inability to specifically ascribe the neural activity patterns we observe to changes in strategic decision-making behavior between the human–human and human–computer conditions. While it was our primary goal to explore changes in neural activity dependent on differences in decision-making when playing against social vs. non-social opponents in an ecologically valid context, we cannot rule out that the mere knowledge that one is playing against a human rather than computer opponent could impact neural activity. We also cannot rule out that other behavioral variables that we were not able to measure, such as gaze behavior during the task, caused the observed differences in neural activity. Finally, familiarity between subjects could be a potential limitation of our study, as differences in familiarity between human and computer opponents could potentially drive some of our observed results. Future experiments can address these issues. First, a condition can be added to our current paradigm in which subjects play against a computer designed to react to the subjects’ responses. This would more closely recapitulate gameplay against a human opponent, and the results could indicate whether the mere knowledge that one is playing against a human opponent is sufficient to drive the results we observe in the absence of differences in interactive behavior. Including dual eye-tracking in the paradigm can further explore how differences in gaze patterns could be driving results. Finally, including only unfamiliar participants can fully rule out the contribution of familiarity differences to our results.

Our study starts to characterize the coordinated neural circuitry underlying interpersonal interaction during competition. We observed that AG, a component of the canonical TPJ, functionally connects with SCA and dlPFC within brains, while similar structures in addition to FG and SS are synchronous across two interacting partners. These findings indicate the intriguing possibility that while coherence across brains involving TPJ corresponds to the updating of social information regarding a human opponent, functional connectivity with prefrontal centers within brains underlies the updating of applied strategy dependent upon social information. Thus, our novel dual-brain paradigm begins to reveal a concerted neural circuitry active during competitive social situations.

## Author Contributions

MP, SC, and JH designed the study. MP and JAN performed the experiments. MP, XZ, and JH analyzed the data and wrote the paper.

## Conflict of Interest Statement

The authors declare that the research was conducted in the absence of any commercial or financial relationships that could be construed as a potential conflict of interest.
