# Supplementary material for: Distributed Neural Activity Patterns during Human-to-Human Competition
Source: Front Hum Neurosci. 2017 Nov 23;11:571. doi: 10.3389/fnhum.2017.00571 (PMC5703701; doi:10.3389/fnhum.2017.00571)
Supplement: Supplementary file 1 [file Data_Sheet_1.pdf]

Supplemental Information

Distributed neural activity patterns during human-to-human competition

Matthew Piva[1], Xian Zhang[2], Adam Noah[2], Steve W. C. Chang[1,3,4], Joy Hirsch[1,2,4,5,6]

Affiliations

1 Interdepartmental Neuroscience Program, Yale University School of Medicine, New Haven, CT 06510

2 Department of Psychiatry, Yale University School of Medicine, New Haven, CT 06510

3 Department of Psychology, Yale University, New Haven, CT 06520

4 Department of Neuroscience, Yale University School of Medicine, New Haven, CT 06510

5 Department of Comparative Medicine, Yale University School of Medicine, New Haven, CT 06510

6 Department of Medical Physics and Biochemistry, University College London, UK

Included are:

Supplemental Figure and Legend

Supplemental Table and Legend

Supplemental References

Supplemental Figure and Legend

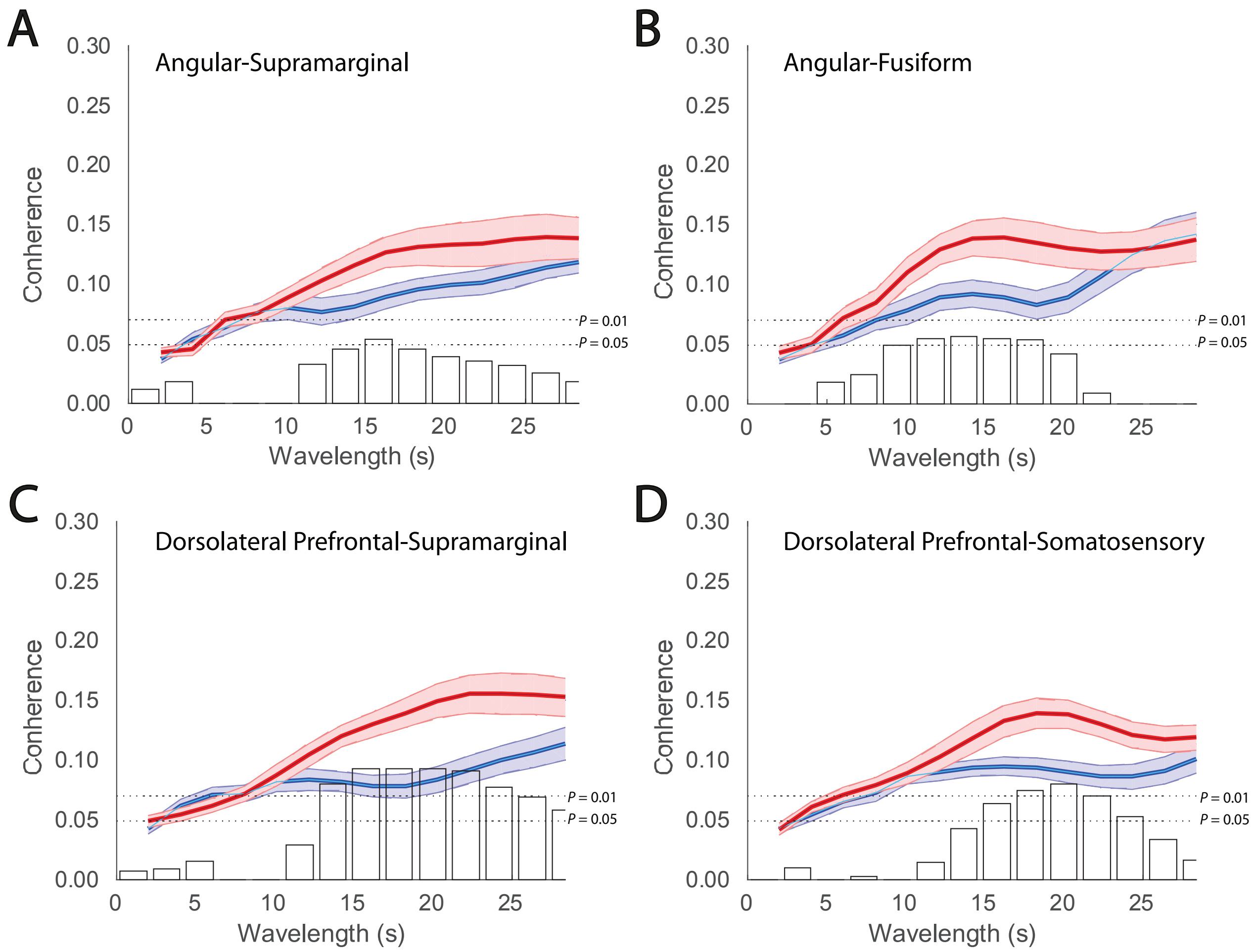

Figure S1. Across-brain coherence varies as a function of condition between distinct neural structures even when only subjects who were complete strangers are included in the analysis. Signal coherence between subjects (y-axis) is plotted against the wavelength (x-axis) for the human-human (red) and the human-computer (blue) conditions (shaded areas: ±1 SEM). Bar graphs indicate the calculated *t*–values for the contrast between the two conditions for each of the wavelength values. The upper horizontal dashed line indicates $P = 0.01$ and the lower line indicates $P = 0.05$, as determined via permutation test. Across-brain coherence is shown between **(A)** angular gyrus and supramarginal gyrus (Partners: $t(19) = 2.24$, P = 4E-02), **(B)** angular gyrus and fusiform gyrus (Partners: $t(19) = 2.33$, P = 3E-02), **(C)** dorsolateral prefrontal cortex (BA46) and supramarginal gyrus (Partners: $t(25) = 4.60$, P = 1E-04), and **(D)** dorsolateral prefrontal cortex (BA46) and somatosensory cortex (Partners: $t(30) = 3.12$, P = 4E-03). Degrees of freedom vary because the number of usable channels differed with some subjects due to anatomical variability related to head size and bad channels.

Supplemental Table and Legend

Table S1. Channels, group-averaged coordinates, anatomical regions, and atlas-based probabilities*

Channel number	MNI Coordinates X	Y	Z	Anatomical Region	BA	Probability
1	56.83	-20.39	48.61	Primary Somatosensory Cortex	1	0.38
				Primary Somatosensory Cortex	3	0.38
				Primary Motor Cortex	4	0.24
2	61.63	-33.70	37.97	Primary Somatosensory Cortex	2	0.18
				Supramarginal Gyrus, part of Wernicke's Area	40	0.82
3	59.36	-7.23	37.74	Primary Somatosensory Cortex	1	0.06
				Primary Somatosensory Cortex	3	0.19
				Primary Motor Cortex	4	0.28
				Pre- and Supplementary Motor Cortex	6	0.14
				Subcentral Area	43	0.33
4	20.35	47.41	47.40	Dorsolateral Prefrontal Cortex	9	1.00
5	50.56	-73.06	13.86	V3	19	0.46
				Fusiform Gyrus	37	0.20
				Angular Gyrus, part of Wernicke's Area	39	0.34
6	61.88	-47.51	17.48	Middle Temporal Gyrus	21	0.25
				Superior Temporal Gyrus	22	0.75
				Fusiform Gyrus	37	0.01
7	63.26	-21.52	26.13	Primary Somatosensory Cortex	2	0.82
				Superior Temporal Gyrus	22	0.10
				Subcentral Area	43	0.07
				Retrosubicular Area	48	0.01
8	60.41	2.82	23.29	Primary Motor Cortex	4	0.01
				Pre- and Supplementary Motor Cortex	6	0.59
				Subcentral Area	43	0.41
9	53.37	27.75	20.58	Pars Opercularis, part of Broca's Area	44	0.02
				Pars Triangularis, part of Broca's Area	45	0.98
10	36.29	48.59	32.06	Dorsolateral Prefrontal Cortex	9	0.18
				Pars Triangularis, part of Broca's Area	45	0.04
				Dorsolateral Prefrontal Cortex	46	0.78

11	57.83	-59.56	-2.98	Fusiform Gyrus	37	1.00
12	64.93	-35.49	9.08	Superior Temporal Gyrus	22	1.00
13	63.38	-9.54	12.93	Superior Temporal Gyrus	22	0.62
				Subcentral Area	43	0.28
				Retrosubicular Area	48	0.11
14	56.74	13.53	5.83	Pre- and Supplementary Motor Cortex	6	0.17
				Temporopolar Area	38	0.15
				Pars Opercularis, part of Broca's Area	44	0.14
				Pars Triangularis, part of Broca's Area	45	0.01
				Retrosubicular Area	48	0.54
15	45.40	46.57	17.01	Pars Triangularis, part of Broca's Area	45	0.44
				Dorsolateral Prefrontal Cortex	46	0.56
16	22.68	61.13	29.80	Dorsolateral Prefrontal Cortex	9	0.26
				Frontopolar Area	10	0.47
				Dorsolateral Prefrontal Cortex	46	0.26
17	61.83	-47.40	-9.79	Inferior Temporal Gyrus	20	0.36
				Fusiform Gyrus	37	0.64
18	65.65	-23.10	-4.17	Middle Temporal Gyrus	21	0.94
				Superior Temporal Gyrus	22	0.06
19	61.23	-2.33	-7.83	Middle Temporal Gyrus	21	0.89
				Temporopolar Area	38	0.02
				Retrosubicular Area	48	0.09
20	51.22	37.68	1.78	Pars Triangularis, part of Broca's area	45	0.82
				Dorsolateral Prefrontal Cortex	46	0.18
21	34.28	61.15	15.32	Frontopolar Area	10	0.69
				Dorsolateral Prefrontal Cortex	46	0.31
22	-53.95	-18.88	47.90	Primary Somatosensory Cortex	1	0.09
				Primary Somatosensory Cortex	3	0.60
				Primary Motor Cortex	4	0.30
				Pre- and Supplementary Motor Cortex	6	0.01

23	-16.15	48.24	46.76	Dorsolateral Prefrontal Cortex	9	1.00
24	-56.34	-7.24	37.61	Primary Somatosensory Cortex	1	0.02
				Primary Somatosensory Cortex	3	0.17
				Primary Motor Cortex	4	0.37
				Pre- and Supplementary Motor Cortex	6	0.14
				Subcentral Area	43	0.30
25	-59.27	-32.34	36.98	Primary Somatosensory Cortex	2	0.46
				Supramarginal Gyrus, part of Wernicke's Area	40	0.54
26	-32.93	49.10	31.93	Dorsolateral Prefrontal Cortex	9	0.15
				Pars Triangularis, part of Broca's Area	45	0.01
				Dorsolateral Prefrontal Cortex	46	0.84
27	-50.21	27.57	20.96	Pars Opercularis, part of Broca's Area	44	0.01
				Pars Triangularis, part of Broca's Area	45	0.99
28	-57.56	2.43	23.44	Pre- and Supplementary Motor Cortex	6	0.57
				Subcentral Area	43	0.43
29	-60.69	-20.44	26.54	Primary Somatosensory Cortex	2	0.70
				Superior Temporal Gyrus	22	0.05
				Subcentral Area	43	0.10
				Retrosubicular Area	48	0.14
30	-60.48	-45.93	19.27	Middle Temporal gyrus	21	0.03
				Superior Temporal Gyrus	22	0.97
31	-51.10	-70.19	17.35	V3	19	0.30
				Fusiform Gyrus	37	0.13
				Angular Gyrus, part of Wernicke's Area	39	0.57
32	-17.33	62.01	30.03	Dorsolateral Prefrontal Cortex	9	0.28
				Frontopolar Area	10	0.56
				Dorsolateral Prefrontal Cortex	46	0.16
33	-41.83	47.52	17.33	Pars Triangularis, part of Broca's Area	45	0.38
				Dorsolateral Prefrontal Cortex	46	0.62

34	-54.34	13.25	6.56	Pre- and Supplementary Motor Cortex	6	0.20
				Temporopolar Area	38	0.06
				Pars Opercularis, part of Broca's Area	44	0.15
				Pars Triangularis, part of Broca's Area	45	0.01
				Retrosubicular Area	48	0.58
35	-60.77	-9.69	14.68	Superior Temporal Gyrus	22	0.54
				Subcentral Area	43	0.35
				Retrosubicular Area	48	0.11
36	-62.47	-34.81	9.89	Superior Temporal Gyrus	22	0.99
				Primary and Auditory Association Cortex	42	0.01
37	-57.43	-57.78	0.33	Fusiform Gyrus	37	1.00
38	-28.93	62.53	15.44	Frontopolar Area	10	0.75
				Dorsolateral Prefrontal Cortex	46	0.25
39	-48.28	38.47	2.13	Pars Triangularis, part of Broca's Area	45	0.79
				Dorsolateral Prefrontal Cortex	46	0.21
40	-59.13	-2.55	-6.89	Middle Temporal Gyrus	21	0.74
				Temporopolar Area	38	0.07
				Retrosubicular Area	48	0.19
41	-63.04	-23.25	-3.67	Middle Temporal Gyrus	21	0.94
				Superior Temporal Gyrus	22	0.06
42	-60.25	-46.80	-8.37	Inferior Temporal Gyrus	20	0.36
				Middle Temporal Gyrus	21	0.04
				Fusiform Gyrus	37	0.60

*Atlas references: Mazziotta et al. (2001), Rorden and Brett (2000)
BA: Brodmann’s Area

Table S1. Channels, group-averaged coordinates, anatomical regions, and atlas-based probabilities. Channel numbers corresponding to clusters (as shown in Figure S1) are listed in the left column. Anatomical locations of the channels are determined for each subject by digitization based on standard 10-20 fiduciary markers. Group averaged centroids are based on the Montreal Neurological Institute (MNI) coordinate system (Mazziotta et al., 2001) and listed as x,y,z coordinates where (-) values indicate left hemisphere. Anatomical region labels are indicated as presented by the SPM-fNIRS utility (Ye et al., 2009) based on representation (Rorden and Brett, 2000) of the Damasio stereotaxic atlas (Damasio and Damasio, 1989). Anatomical regions, including Brodmann's Areas (BA) with probabilities of inclusion in the cluster, are shown in the center and right columns.

Supplemental References

Damasio, H., and Damasio, A.R. (1989). *Lesion analysis in neuropsychology.* New York: Oxford University Press.

Mazziotta, J., Toga, A., Evans, A., Fox, P., Lancaster, J., Zilles, K., Woods, R., Paus, T., Simpson, G., Pike, B., Holmes, C., Collins, L., Thompson, P., Macdonald, D., Iacoboni, M., Schormann, T., Amunts, K., Palomero-Gallagher, N., Geyer, S., Parsons, L., Narr, K., Kabani, N., Le Goualher, G., Boomsma, D., Cannon, T., Kawashima, R., and Mazoyer, B. (2001). A probabilistic atlas and reference system for the human brain: International Consortium for Brain Mapping (ICBM). *Philos Trans R Soc Lond B Biol Sci* 356**,** 1293-1322.

Rorden, C., and Brett, M. (2000). Stereotaxic display of brain lesions. *Behav Neurol* 12**,** 191-200.

Ye, J.C., Tak, S., Jang, K.E., Jung, J., and Jang, J. (2009). NIRS-SPM: statistical parametric mapping for near-infrared spectroscopy. *Neuroimage* 44**,** 428-447.
